# DT-Loong: A Digital Twin Simulation Framework for Scalable Data Collection and Training of Humanoid Robots

**DOI:** 10.3390/biomimetics10110725

**Published:** 2025-11-01

**Authors:** Yufei Liu, Yang Li, Jinda Du, Yanjie Rui, Yongyao Li

**Affiliations:** 1 Unmanned Vehicle Research Center, China North Vehicle Research Institute, Beijing 100072, China; liuyufei@openloong.net (Y.L.); yanjie.rui@openloong.net (Y.R.); 2 The National and Local Co-Build Humanoid Robotics Innovation Centre, Shanghai 201203, China; liyang@openloong.net (Y.L.); jindadu00@sjtu.edu.cn (J.D.); 3 Humanoid Robot (Shanghai) Co., Ltd., Shanghai 201203, China; 4 Shanghai Jiao Tong University, Shanghai 200240, China

**Keywords:** humanoid teleoperation, digital twin system, motion capture, bionic-inspired humanoid robot systems

## Abstract

Recent advances in bionic intelligence are reshaping humanoid-robot design, demonstrating unprecedented agility, dexterity and task versatility. These breakthroughs drive an increasing need for large scale and high-quality data. Current data generation methods, however, are often expensive and time-consuming. To address this, we introduce Digital Twin Loong (DT-Loong), a digital twin system that combines a high-fidelity simulation environment with a full-scale virtual replica of the humanoid robot Loong, a bionic robot encompassing biomimetic joint design and movement mechanism. By integrating optical motion capture and human-to-humanoid motion re-targeting technologies, DT-Loong generates data for training and refining embodied AI models. We showcase the data collected from the system is of high quality. DT-Loong also proposes a Priority-Guided Quadratic Optimization algorithm for action retargeting, which achieves lower time delay and enhanced mapping accuracy. This approach enables real-time environmental feedback and anomaly detection, making it well-suited for monitoring and patrol applications. Our comprehensive framework establishes a foundation for humanoid robot training and further digital twin applications in humanoid robots to enhance their human-like behaviors through the emulation of biological systems and learning processes.

## 1. Introduction

Over the past few years, bionic intelligence and humanoid robotics have progressed at an exceptional pace. By mimicking biological morphology, materials and behavioral intelligence, humanoid robotics can yield more naturalistic locomotion, dexterous manipulation and adaptive interaction [1].

However, training such robots hinges on massive and diverse data to unlock advanced intelligent behaviors performed by humanoid robots. Teleoperation, passive observation from human videos, and hand-held grippers [2,3] are three common approaches to obtain reliable training data. While current efforts have made many robot training datasets open-source [4,5], these datasets are often limited to fixed scenarios, tasks or embodiments and are collected with high cost.

Simulation is an alternative to acquiring real-world robot data. It can alleviate issues associated with fixed scenario setups and reliance on physical robots, and thus saving both time and money. However, challenges remain in bridging the sim-to-real gaps [6] for practical applications.

Another limitation of current studies is that most robot platforms are fixed [2], primarily designed for specific table-top manipulation tasks. Consequently, there lacks sufficient data on loco-manipulation [7,8], whole-body control of humanoid robots [9,10,11], and interactions between virtual and real environments [12].

**Figure 1:**
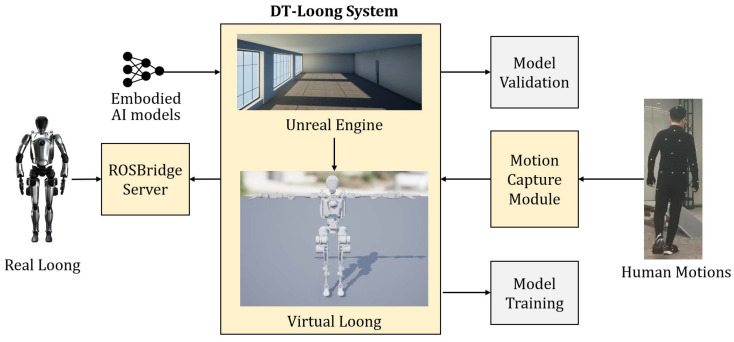
System overview. DT-Loong is a novel framework that leverages a motion capture module to record human motion and map it into Loong’s joint space. The digital twin replica then updates and outputs joint space states and sensor feedback, supporting model training and performance evaluation. DT-Loong also enables real-world implementations such as monitoring tasks and serves as an adaptable testbed for optimizing embodied AI models, making it a versatile tool for both research and practical deployment.

In this paper, we present DT-Loong, a multi-functional digital twin framework for humanoid robots Figure 1. By replicating real-world environments and the bionic robot Loong equipped with an IMU (Inertia Measurement Unit) and visual sensors, we build a digital environment using UE5 (Unreal Engine 5). A MoCap (Motion Capture) system with a delicately designed retargeting algorithm is used to convert human data into humanoid robot data for further training. Beyond data collection, DT-Loong also serves as a platform to test the applicability of embodied AI models, promoting the development of more bio-inspired intelligence. In addition, when Loong is deployed in monitoring and patrolling scenarios, DT-Loong can predict the Loong ’s actions and alert human operators of a surveillance system.

In summary, the main contributions of this paper are:

- We propose DT-Loong, a digital twin system capable of low-cost, large-scale, and full-body humanoid robot data collection from human visual demonstrations, supporting both model optimization and practical monitoring applications.
- Through the design of a retargeting algorithm and visualization techniques, we demonstrate that the data generated by DT-Loong is of high quality and strong usability, and can facilitate the training of more naturalistic robot intelligence.
- We develop an alarming and anomaly detection framework that can trigger alerts in real-time, assisting human operators in surveillance scenarios.

## 2. Related Work

### 2.1. Data Collection for Humanoid Robots

Teleoperation is the most widely adopted method for imitation learning of robots. By deploying VR/AR controllers [13,14,15], MoCap suits [16], exoskeletons [17], haptic force feedback [18], etc., human knowledge and experience can be transferred to remotely controlled robots. Major contributions in this field include ALOHA series [2,19,20], OpenTelevision [21], HumanPlus [22], BiDex [23].

While task-space and upper-body teleoperation have attracted more academic attention, whole-body teleoperation remains significantly more challenging. These difficulties stem from differences in kinematics, the need to fully exploit the shared morphology between humans and robots and the tradeoff between similarity and feasibility [24]. Amartya Purushottam [25] develops a whole-body teleoperation frame work and a kinematic retargeting strategy for a wheeled humanoid. H2O [26] introduces a learning-based real-time whole-body teleoperation approach for a full-sized humanoid robot using an RGB camera, and OmniH2O [27] extends this approach to achieve high-precision dexterous loco-manipulation.

MoCap systems serve as a powerful tool to transfer the natural styles and synergies of human behaviors to humanoid robots. DexCap [28] provides an effective way to collect data for dexterous manipulation using a portable MoCap system. Building upon previous research, our approach integrates a MoCap device with a retargeting algorithm to enable whole-body teleoperation of a fully-sized bionic humanoid, Loong, designed with a biomimetic configuration. The resulting comprehensive framework is well-suited for large-scale humanoid data collection and model training.

### 2.2. Digital Twin in Robotics

The concept of digital twin was first brought up by Michael Grieves [29]. Digital twin systems have been widely used in industrial applications [30]; however, research on their use in humanoid robotics remains limited. RoboTwin [31] creates a diverse data generator for dual-arm robots using 3D generative foundation models. DTPAAL [32] demonstrates the capabilities of a digital-twinned Pepper robot in assisting elderly individuals at home. Jon Skerlj [33] proposes a safe-by-design digital twin framework for humanoid service robots.

One important application of digital twin in robotics is system monitoring, where robot path-planning [34] and collision detection play crucial roles. A clothoid approximation approach [35] using Bézier curves is developed to enhance path accuracy and planning efficiency. An efficient approach [36] based on Separating Axis Theorem (SAT) is applied to the real humanoid robot, DRC-HUBO+. Our work presents a vivid replica of a real-world environment and the robot Loong. The system DT-Loong provides a platform to assist human operators in real-world scenarios, including guarding, monitoring, and anomaly detection.

## 3. System Design

### 3.1. Overall Architecture

**Figure 2:**
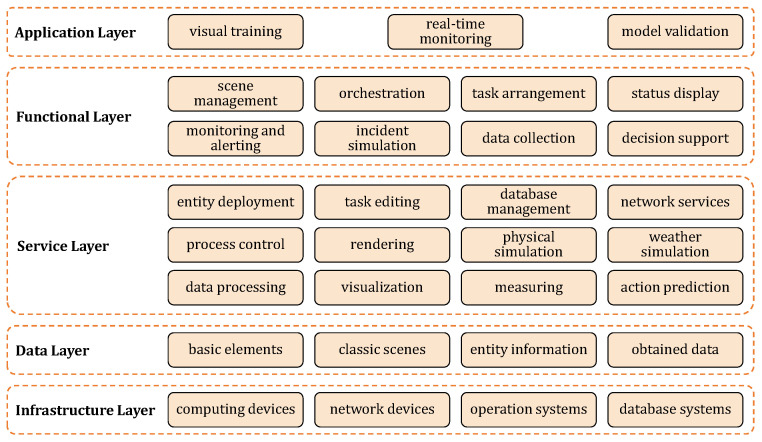
System architecture illustrating the hierarchical layers from infrastructure to application, detailing the core components involved in visual training, real-time monitoring, and model validation.

As shown in Figure 2, the DT-Loong system is composed of five layers. The upper two layers deal with higher-level functions and applications of the system, while the lower layers focus on core services and infrastructure.

The application layer represents the system’s usage, including data collection from visual demonstrations, real-time monitoring for patrol tasks, and algorithm validation to enhance humanoid behaviors. The functional layer manages essential features such as scenario setup, orchestration, task arrangement, etc. The service layer provides core system services, establishing foundational capabilities related to entities, processes, simulations, and associated functionalities. The data layer is responsible for managing data resources, and the infrastructure layer provides the necessary hardware and software environment.

**Figure 3:**
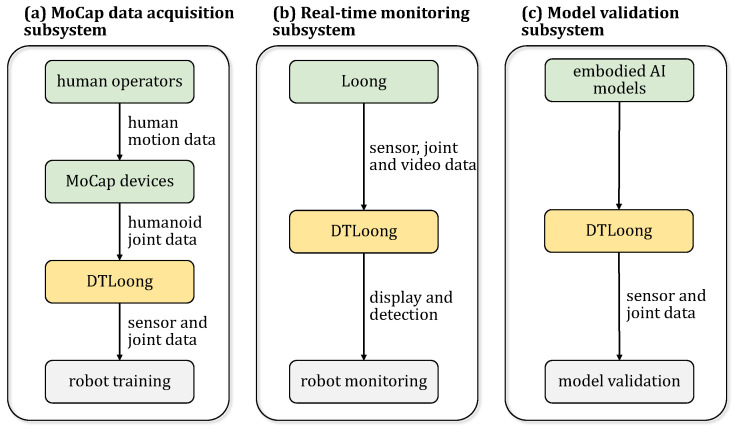
Subsystems of DT-Loong. (**a**) The MoCap data acquisition subsystem collects human motion data and generates corresponding humanoid joint data for robot training. (**b**) The real-time monitoring subsystem monitors robot movements by processing sensor, joint, and video data. (**c**) The model validation subsystem uses the humanoid robot’s data to test and improve AI models.

The overall workflow is depicted in Figure 1. Based on practical applications, DT-Loong can be divided into three subsystems, as shown in Figure 3. This modular design enhances the independence of each subsystem and improves the system’s maintainability and scalability.

### 3.2. MoCap Data Acquisition Subsystem

Targeted at preparing data for humanoid imitation learning, the MoCap data acquisition subsystem employs optical motion capture technology to obtain Cartesian-space coordinates that represent human body motions, which are then mapped into the joint-space coordinates of Loong. After integrating these data into DT-Loong, the system outputs IMU data, camera data, and humanoid joint data in hdf5 files for subsequent robot model training.

### 3.3. Real-Time Monitoring Subsystem

In commercial buildings, deploying physical robots such as Loong for patrolling and monitoring provides a practical alternative to human guards. The real-time monitoring subsystem collects sensor data, joint data, and video feeds, which are transmitted to DT-Loong. Within DT-Loong, the virtual replica mirrors Loong’s movements, allowing the system to display its status and send warnings upon detection of falls, collisions, or immobilization.

### 3.4. Model Validation Subsystem

For widely used humanoid embodied AI models such as VLMs [37] and VLAs [38], the model validation subsystem works as a cost-effective platform to evaluate their applicability within the high-fidelity simulation environment of DT-Loong. The system can generate simulated sensor data using the *USceneCaptureComponent* in UE5. Furthermore, the model under test can control the virtual Loong perform movements and produce pose and displacement data, enabling performance validation through intuitive observation.

## 4. Implementation

### 4.1. Digital Twin Environment Establishment

**Figure 4:**
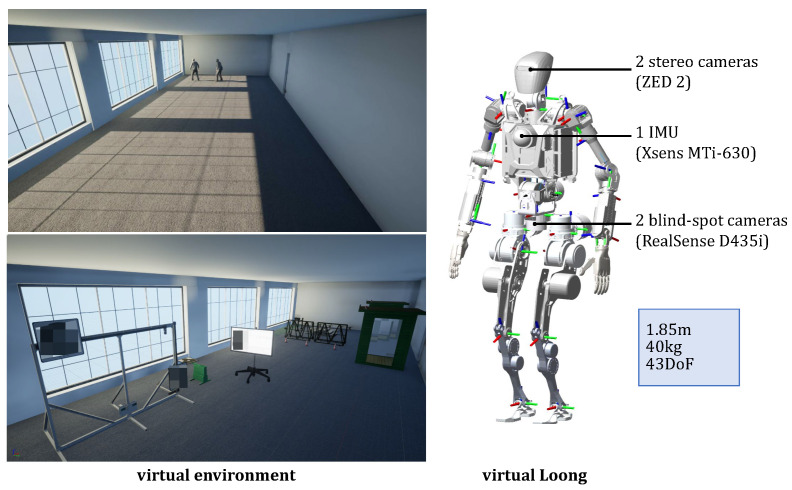
Virtual environment and humanoid.

The virtual environment and Loong is shown in Figure 4. To establish basic scenes for the practical application of DT-Loong, we apply UE5 together with Maya and Blender to construct fundamental indoor and outdoor environments incorporating weather condition simulation, high-fidelity entities, and simulation task setups. For the primary application of patrolling and monitoring, in addition to Loong, we build entities that represent a commercial hall, security devices and potential intruders.

Loong https://www.openloong.org.cn/cn/documents/robot/product_introduction (accessed on 13 October 2025) is an open-source bionic humanoid robot with 43 degrees of freedom (DoF), a height of 1.85 m, and a weight of 40 kg. Featuring a human-like joint design focused on the hip, waist, knee, and ankle regions, Loong is capable of fast walking, agile obstacle avoidance, stable uphill/downhill traversal, and robust impact resistance. To digitally represent Loong, we simplify the full-scale 3D model by reducing the number of faces and vertices and replicate its sensor setups. Specifically, two stereo cameras (ZED 2) are placed at Loong’s eyes, two blind-spot cameras (RealSense D435i, Intel Corporation, Santa Clara, CA, USA) are mounted at the middle of the body, and an IMU (Xsens MTi-630, Xsens, Enschede, The Netherlands) is positioned in the chest area.

In addition to physical simulation, we develop task-specific services and functions. PostgreSQL is employed for data acquisition, processing, storage, and database management. We also implement entity and task management, special event simulation, as well as visualization and warning mechanisms.

Our methods for addressing key challenges, such as human-to-humanoid retargeting and task scheduling, are detailed in Section 4.2 and Section 4.3. The three subsystems outlined in Section 3 rely on the integration and coordination of these fundamental components.

### 4.2. Robot Retargeting

Data acquisition is the primary objective of DT-Loong. We carefully design a MoCap system combining optical markers and an IMU to capture full-body human motion. A motion retargeting algorithm then maps these movements to the humanoid robot, considering joint limits and kinematics, ensuring that the motions are robot-executable and the resulting data are suitable for training embodied AI models.

**Figure 5:**
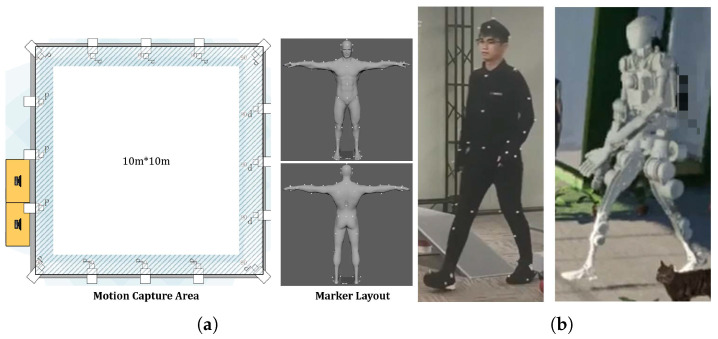
MoCap system. (**a**) MoCap setup. (**b**) MoCap demonstration.

As depicted in Figure 5, our MoCap system is set up in a 100 m^2^ square room, with sixteen MC1300 infrared cameras mounted on the truss around the ceiling. We place 53 markers on the human body to capture motion data. The data collected from these markers are then passed to the MoCap module for human-to-humanoid retargeting.

To convert human motion data into humanoid joint state parameters, we adopt a hierarchical QP (PGQO (Priority-Guided Quadratic Optimization)) formulation. Each task is defined by equality and/or inequality constraints with respect to the decision variable *x*. Tasks sharing the same priority *i* are vertically stacked into a composite task $T_{i}$:

(1) $$T_{i}:\left\{\begin{array}{c} A_{i}x\approx b_{i},\\ D_{i}x\leq f_{i}, \end{array}\right.$$

Here, $A_{i}x\approx b_{i}$ denotes equality-type tracking objectives that will be enforced in a least-squares sense when forming the QP at priority *i*, and $D_{i}x\leq f_{i}$ denotes inequality constraints for the same priority. The optimization variable *x* represents joint angular velocities. Variables A, b, D, f are defined in Table 1. Specifically, $J_{1}$ is the Jacobian of end-effector pose error, $J_{2}$ is the Jacobian of body-segment direction, and $J_{3}$ is the Jacobian of the manipulability index [39]. The vector *b* is the PD term associated with pose error, and *I* is the identity matrix.

#### 4.2.1. Single-Priority QP

At priority *i*, we solve the following weighted least-squares QP with nonnegative slack $v_{i}$:

(2) $$\begin{array}{cc} \min_{x,v_{i}} & \frac{1}{2}\lambda_{i}∥A_{i}x-b_{i}{∥}^{2}+\frac{1}{2}\mu_{i}{∥v_{i}∥}^{2}\\ s.t. & D_{i}x-f_{i}\;\leq \;v_{i},\\ \; & v_{i}\;\geq \;0, \end{array}$$

with scalar weights $\lambda_{i},\mu_{i}>0$. The slack $v_{i}$ vanishes whenever the inequalities are feasible; if not, $v_{i}>0$ provides the minimal relaxation required to retain feasibility.

#### 4.2.2. Hierarchical Optimization

To guarantee task prioritization, we solve a sequence of QPs. At priority *i*, we optimize over the set of optimal solutions of all higher priorities:

(3) $$\begin{array}{cc} h_{i}\left(x_{k}^{*},v_{k}^{*}\right)=\arg\min_{x,\;v}\; & ∥A_{i}x-b_{i}{∥}_{W_{i}}^{2}+{∥v∥}_{V_{i}}^{2}\\ s.t. & D_{k}x-f_{k}\leq v_{k}^{*},\;\forall \;k<i,\\ \; & A_{k}\left(x-x_{k}^{*}\right)=0,\;\forall \;k<i,\\ \; & v\geq 0, \end{array}$$

where $\left(x_{k}^{*},v_{k}^{*}\right)$ is the solution at priority k<i.

#### 4.2.3. Canonical QP Form

Let $\xi_{i}=\left(z_{i},v_{i}\right)$. Then

(4) $$\min_{\xi_{i}}\;\;\frac{1}{2}\;\xi_{i}^{⊤}H_{i}\xi_{i}\;+\;g_{i}^{⊤}\xi_{i}\;s.t.\;\;{\tilde{D}}_{i}\xi_{i}\leq {\tilde{f}}_{i},$$

with

(5) $$H_{i}=\left[\begin{array}{cc} Z_{i-1}^{⊤}{\tilde{A}}_{i}^{⊤}{\tilde{A}}_{i}\;Z_{i-1} & 0\\ 0 & V_{i}^{⊤}V_{i} \end{array}\right],\;g_{i}=\left[\begin{array}{c} Z_{i-1}^{⊤}{\tilde{A}}_{i}^{⊤}\left({\tilde{A}}_{i}x_{i-1}^{*}-{\tilde{b}}_{i}\right) \end{array}\right],$$

(6) $${\tilde{D}}_{i}=\left[\begin{array}{cc} D_{1}Z_{i-1} & 0\\ \vdots & \vdots \\ D_{i-1}Z_{i-1} & 0\\ D_{i}Z_{i-1} & I\\ 0 & -I \end{array}\right],\;{\tilde{f}}_{i}=\left[\begin{array}{c} f_{1}-D_{1}x_{i-1}^{*}+v_{1}^{*}\\ \vdots \\ f_{i-1}-D_{i-1}x_{i-1}^{*}+v_{i-1}^{*} \end{array}\right].$$

#### 4.2.4. Null-Space Recursion

To preserve higher-priority equalities, we compute the null-space basis recursively:

(7) $$Z_{0}=I,\;A_{i,\mathrm{pre}}=A_{i}Z_{i-1},\;Z_{i}=Z_{i-1}\;N\left(A_{i,\mathrm{pre}}\right),$$

where N(·) returns an orthonormal basis of the null space.

### 4.3. Task Scheduling

We design a task scheduling system that manages tasks based on priority. It features task parameter editing, task instance execution, task lifecycle management, cross-task scheduling, and script management.

There are three sources of tasks: coordination tasks, which directly interrupt current tasks; to-do scenario tasks, which can be selected from the queue or suspended pool for execution; and pre-configured autonomous actions. We manage these tasks to enable unified scheduling and state transitions.

Additionally, we perform serialization and deserialization to streamline and accelerate the process. The event serialization function efficiently organizes and encodes complex event data structures generated by the event editing module, transforming them into a serialized format for easy storage. Specifically, the events are encoded into JSON string format, with RapidJSON used for encoding and parsing the JSON. During the actual training phase, the serialized event information is decoded and restored. Through deserialization, the original event model and attributes are reconstructed, ensuring that the events are executed precisely within their preset temporal logic framework.

## 5. Experimental Results

### 5.1. Scenario Setup

To evaluate the applicability of DT-Loong, we design multiple scenarios to rigorously test its performance. These include an indoor environment where Loong is deployed for patrolling and monitoring, as illustrated in Figure 4, and an outdoor setting where Loong is applied to capture human motions and perform Tai Chi exercises, as depicted in Figure 8. These diverse environments enable a practical assessment of the system’s adaptability and robustness across a variety of real-world contexts.

### 5.2. Status Visualization

**Figure 6:**
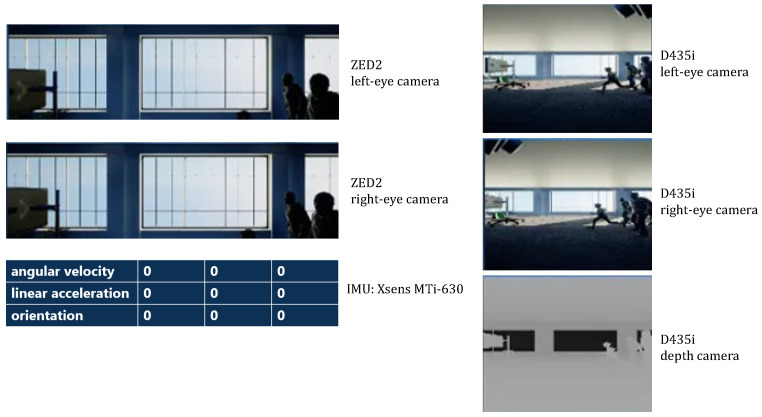
Sensor board displaying camera and IMU information (demo). It aggregates live views from the ZED2 stereo RGB camera (left/right) and the Intel RealSense D435i (left/right and depth), together with IMU readouts–angular velocity, linear acceleration, and orientation—from an Xsens MTi-630.

**Figure 7:**
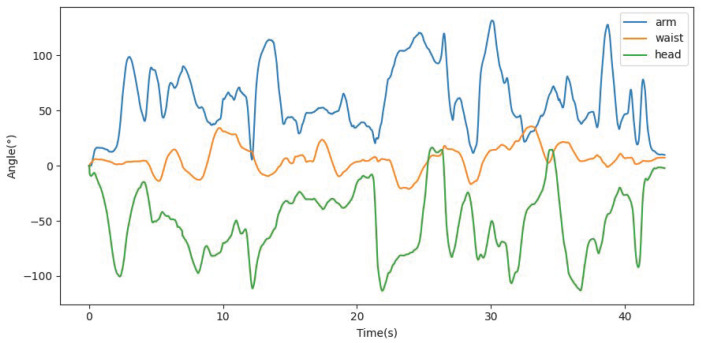
Sample joint angle curves from Tai Chi practices locating at Loong ’s arm, waist, and head.

**Figure 8:**
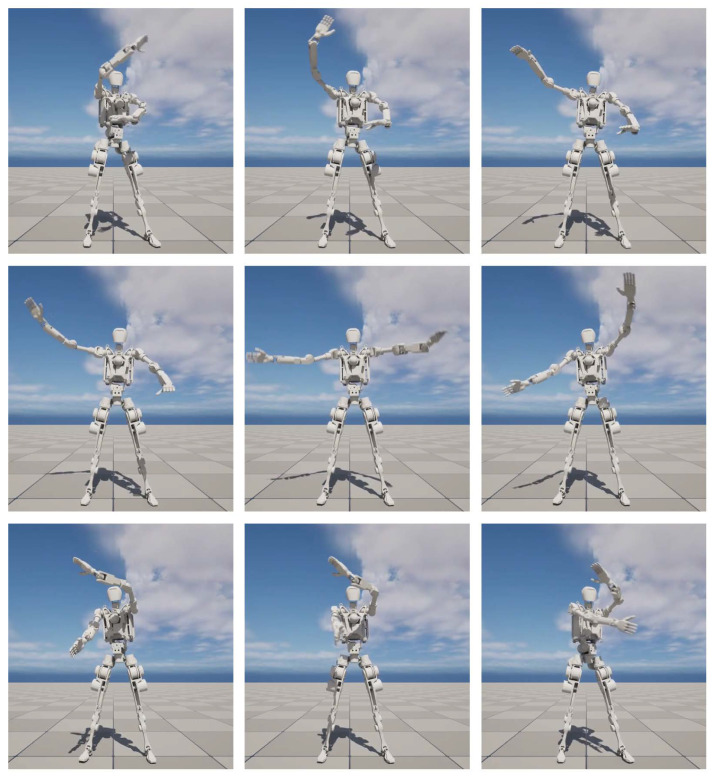
Movements of the virtual Loong performing Tai Chi. It illustrates natural, human-like actions produced by our retargeting algorithm.

DT-Loong provides a user-friendly interface that allows users to easily observe sensor data, humanoid movements and the virtual Loong’s interactions with the environment. Figure 6 presents the observation window, displaying feeds from the two stereo cameras, two blind-spot cameras, and data gathered from the IMU. Figure 7 shows three sample joint angle curves of Loong, which are captured from a video demo of the virtual Loong practicing Tai Chi, as shown in Figure 8. These visualizations facilitate a clearer understanding of the complex dynamics involved in the Tai Chi movement sequences.

### 5.3. Real-World Applications

**Figure 9:**
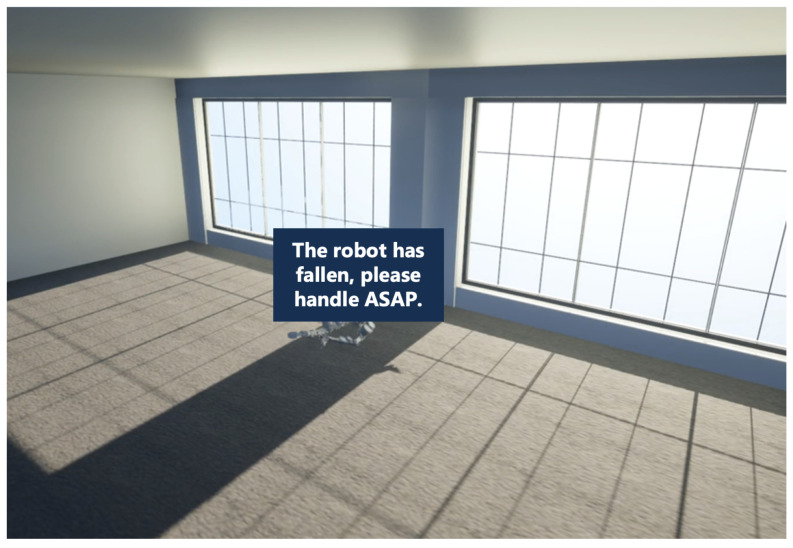
System alert.

**Figure 10:**
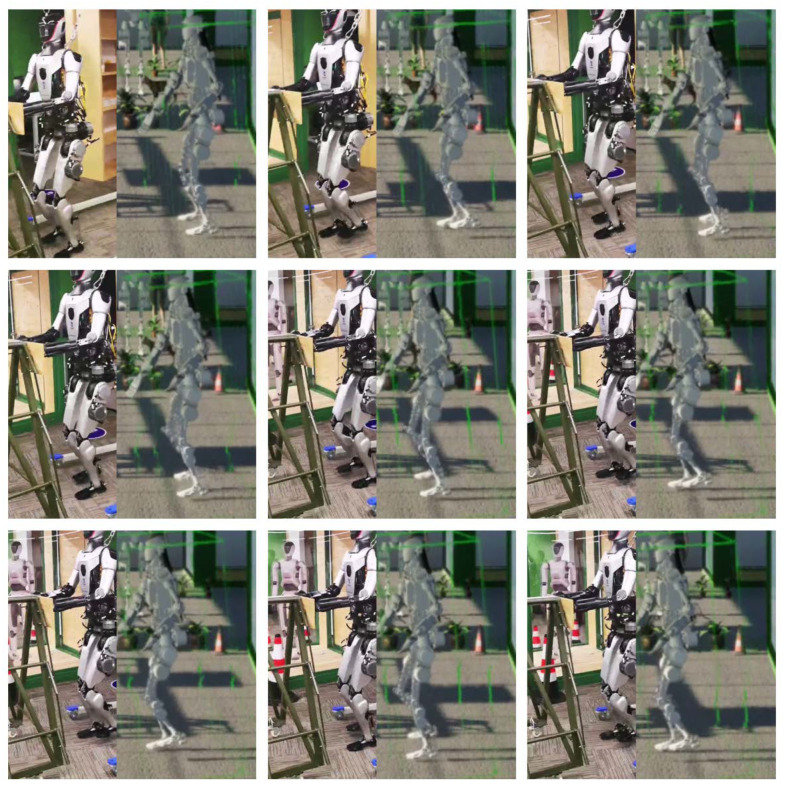
Application of the monitoring subsystem.

Indoor spaces such as banks, offices and shopping malls typically require a large number of guards to maintain order and ensure safety. Deploying Loong in these environments with the support of DT-Loong provides a practical alternative to reduce human labor. Once the virtual Loong falls or collides with an object (such as an intruder), or remains static for an extended period, our DT-Loong system will send out warnings to alert human operators to handle the situation, as shown in Figure 9. Figure 10 illustrates the virtual representation of Loong mimicking the walking pose of its physical counterpart. It demonstrates the effectiveness of our monitoring subsystem and highlights its applicability in practical surveillance tasks.

## 6. Discussion and Conclusions

In this study, we propose a novel digital twin framework for humanoid data collection, status monitoring, and algorithm evaluation. By incorporating an optical MoCap system and a hierarchical quadratic programming method, our approach addresses the challenges of large-scale full-body humanoid data collection in a cost-effective manner. While the monitoring system currently emphasizes Loong’s locomotion, it does not yet include mapping of human hand motions. Another limitation is that, as the current work focuses on system functionality and practical applications, quantitative evaluations of data quality and mapping performance are not fully addressed. Future efforts will be put into enhancing system responsiveness through improved data compression and networking techniques. Additionally, by developing a more fine-grained physical simulation mechanism–improving simulation speed, diversity and accuracy, DT-Loong could serve as a comprehensive digital twin platform for training, evaluating, and deploying bionic humanoid robots across diverse operational scenarios.

## Figures and Tables

**Table 1:** Optimization tasks.

Tasks	Priority	A	b	D	f
Upper body end-effectors 6D pose tracking	0	$J_{1}$	$K_{p}Err+K_{i}\int_{0}^{t}Err\left(r\right)\;dr$	$\left[\begin{array}{c} I\\ -I \end{array}\right]$	$\left[\begin{array}{c} {\dot{q}}_{max}\\ -{\dot{q}}_{min} \end{array}\right]$
Whole body segment orientation tracking	1	$J_{2}$	$K_{pi}Err_{i}+K_{ii}\int_{0}^{t}Err_{i}\left(r\right)\;dr$	none	none
Manipulability index	1	$J_{3}$	$K_{p}Err_{i}+K_{i}\int_{0}^{t}Err_{i}\left(r\right)\;dr$	none	none

## Data Availability

The original contributions presented in this study are included in the article. Further inquiries can be directed to the corresponding authors.
